# Empagliflozin Reduces Renal Hyperfiltration in Response to Uninephrectomy, but Is Not Nephroprotective in UNx/DOCA/Salt Mouse Models

**DOI:** 10.3389/fphar.2021.761855

**Published:** 2021-12-21

**Authors:** Philipp Tauber, Frederick Sinha, Raffaela S. Berger, Wolfram Gronwald, Katja Dettmer, Michaela Kuhn, Maximilian Trum, Lars S. Maier, Stefan Wagner, Frank Schweda

**Affiliations:** ^1^ Institute of Physiology, University of Regensburg, Regensburg, Germany; ^2^ Institute of Functional Genomics, University of Regensburg, Regensburg, Germany; ^3^ Institute of Physiology, University of Würzburg, Würzburg, Germany; ^4^ Department of Internal Medicine II, University Hospital Regensburg, Regensburg, Germany

**Keywords:** SGLT2 inhibition, empagliflozin (EMPA), hyperfiltration, UNx/DOCA/salt model, nephroprotection

## Abstract

Large-scale clinical outcome studies demonstrated the efficacy of SGLT2 inhibitors in patients with type II diabetes. Besides their therapeutic efficacy in diabetes, significant renoprotection was observed in non-diabetic patients with chronic kidney disease (CKD), suggesting the existence of glucose-independent beneficial effects of SGLT2 inhibitors. However, the relevant mechanisms by which SGLT2 inhibition delays the progression of renal injury are still largely unknown and speculative. Previous studies showed that SGLT2 inhibitors reduce diabetic hyperfiltration, which is likely a key element in renoprotection. In line with this hypothesis, this study aimed to investigate the nephroprotective effects of the SGLT2 inhibitor empagliflozin (EMPA) in different mouse models with non-diabetic hyperfiltration and progressing CKD to identify the underlying diabetes-independent cellular mechanisms. Non-diabetic hyperfiltration was induced by unilateral nephrectomy (UNx). Since UNx alone does not result in renal damage, renal disease models with varying degrees of glomerular damage and albuminuria were generated by combining UNx with high NaCl diets ± deoxycorticosterone acetate (DOCA) in different mouse strains with and without genetic predisposition for glomerular injury. Renal parameters (GFR, albuminuria, urine volume) were monitored for 4–6 weeks. Application of EMPA via the drinking water resulted in sufficient EMPA plasma concentration and caused glucosuria, diuresis and in some models renal hypertrophy. EMPA had no effect on GFR in untreated wildtype animals, but significantly reduced hyperfiltration after UNx by 36%. In contrast, EMPA did not reduce UNx induced hyperfiltration in any of our kidney disease models, regardless of their degree of glomerular damage caused by DOCA/salt treatment. Consistent with the lack of reduction in glomerular hyperfiltration, EMPA-treated animals developed albuminuria and renal fibrosis to a similar extent as H_2_O control animals. Taken together, the data clearly indicate that blockade of SGLT2 has the potential to reduce non-diabetic hyperfiltration in otherwise untreated mice. However, no effects on hyperfiltration or progression of renal injury were observed in hypervolemic kidney disease models, suggesting that high salt intake and extracellular volume might attenuate the protective effects of SGLT2 blockers.

## Introduction

Over the last years, inhibitors of the renal sodium-glucose transporter SGLT2, a new class of antidiabetic drugs, have demonstrated their beneficial effect on the progression of diabetic nephropathy in several large-scale clinical outcome trials (Zinman et al., 2015; Wanner et al., 2016; Neal et al., 2017; Wiviott et al., 2019). Since these initial clinical trials were conducted exclusively in patients with type II diabetes, it remained unclear whether the nephroprotective effects due to SGLT2 inhibition could be translated to non-diabetic chronic kidney disease (CKD). Indeed, several lines of evidence indicate that the beneficial effects of SGLT2 inhibitors cannot be explained by a reduction of blood glucose levels alone. For example, post hoc analyses of clinical trials revealed that renal protection by empagliflozin (EMPA) and canagliflozin was independent of HbA_1C_ levels (glycated hemoglobin) before and during therapy with SGLT2 inhibitors (Heerspink et al., 2017; Cooper et al., 2019). Moreover, the immediate reduction in diabetic hyperfiltration after initiation of therapy with EMPA and the reversibility of this effect after discontinuation suggested that rapid functional effects, rather than structural changes, underlie the renal benefits of SGLT2 inhibition (Wanner et al., 2016). In fact, more recently, the DAPA-CKD trial confirmed these speculations by demonstrating that the SGLT2 inhibitor dapagliflozin significantly lowered the risk of glomerular filtration rate (GFR) decline, end-stage kidney disease, and death from renal cause in patients with CKD, regardless of their glycemic status (Heerspink et al., 2020). While the therapeutic potential of SGLT2 inhibitors for patients with diabetes and CKD is undisputed, the underlying mechanisms of renal protection remain unclear. The current hypotheses involve metabolic aspects such as changes in lipid metabolism and enhanced ketone body production (Thomas and Cherney, 2018; Szekeres et al., 2021), as well as hemodynamic (Thomson and Vallon, 2021), and hypoxic effects (Packer, 2021) of SGLT2 inhibition. Moreover, the immediate reduction in diabetic glomerular hyperfiltration appears to be a common event in patients on SGLT2 inhibitors and is likely critical for long-term maintenance of GFR and prevention of progressive kidney damage (Kohan et al., 2016; Wanner et al., 2016; Perkovic et al., 2018; Perkovic et al., 2019).

This so-called hyperfiltration or tubular hypothesis is based on the fact that in a diabetic metabolic state, a hyperreabsorption of glucose and NaCl by SGLT2 occurs in the early proximal tubule, which subsequently leads to a decrease in the luminal NaCl concentration perceived at the macula densa. The cells of the macula densa are functionally coupled to the vascular pole of the same nephron (tubuloglomerular feedback mechanism, TGF). Low NaCl at the macula densa attenuates the TGF response, leading to dilatation of the vas afferens, which increases intraglomerular pressure and explains diabetic hyperfiltration (Vallon and Thomson, 2020). Micropuncture experiments in diabetic rats showed that under SGLT2 inhibition, which reduces proximal hyperreabsorption of NaCl and glucose, the TGF system is reactivated, leading to a reduction in glomerular capillary pressure (5–8 mm Hg) and decreased diabetic hyperfiltration (−25%) (Thomson and Vallon, 2021). Glomerular hyperfiltration is not limited to diabetic conditions, but can rather be seen as a general compensatory response in progressive renal disease (Brenner et al., 1996; Fattah et al., 2019). Loss of functional nephrons increases glomerular blood flow in the remaining healthy nephrons, resulting in higher single-nephron GFR, which is needed to maintain total kidney GFR constant (Hayslett et al., 1968; Brenner et al., 1972; Denic et al., 2017). However, in the long term, mechanical stress due to high intraglomerular pressure induces damage of the glomerular filtration barrier, causing proteinuria, glomerular sclerosis, and eventually further loss of functional nephrons (Denic et al., 2017; Sharma et al., 2017). Therefore, we speculated that the renal benefits of SGLT2 inhibitors in kidneys with non-diabetic CKD might be the result of a reduction of hyperfiltration in functional nephrons, hereby reducing mechanical stress and glomerular damage. To address this point, this study examined the nephroprotective effect of EMPA in uninephrectomized wildtype mice and four different kidney disease mouse models with varying degrees of glomerular damage/albuminuria, referring to an early clinical study that linked the occurrence of renal benefits by SGLT2 inhibition with the patient’s stage of renal impairment at therapy initiation (Rajasekeran et al., 2018). To generate mouse models with varying severity of glomerular damage, hyperfiltration was induced by unilateral nephrectomy (UNx). Since UNx alone does not result in renal damage, it was combined with a high NaCl diet and/or deoxycorticosterone acetate (DOCA) in wildtype mice or mice with different genetic predispositions for glomerular injury. Based on results of previous studies that demonstrated increased susceptibility to glomerular damage in mice with genetic deletion of the ANP/BNP receptor guanylyl cyclase-A in podocytes (Staffel et al., 2017), either podocyte specific GC-A knockout mice or mice with general deletion of GC-A were used to aggravate renal damage.

## Materials and Methods

### Animals

In this study, we used male mice at the age of 12–16 weeks. The generation of mice with a podocyte-specific (Podo GC-A KO) or global deletion (GC-A KO) of the natriuretic peptide receptor guanylylcyclase-A (GC-A) has been described elsewhere (Lopez et al., 1995; Staffel et al., 2017). C57BL/6J mice were purchased from Charles River (Sulzfeld, Germany). Offspring of homozygous breeder pairs were used throughout the study. Animals had free access to food and water. All experimental procedures were conducted in accordance with the German Animal Welfare Act and approved by the local authorities (government of Lower Franconia, Germany, file number RUF 55.2.2-2532.2-896-13).

### UNx/DOCA/Salt Models

For all models (see overview Table 1), UNx was used to induce non-diabetic hyperfiltration in the remaining kidney. UNx was performed as described in a previous study by Staffel et al. (2017). No increased mortality after UNx intervention was observed.

**TABLE 1:** Description of kidney disease mouse models used in this study. UNx, unilateral nephrectomy; High salt diet, food contained 4% NaCl; DOCA, deoxycorticosterone acetate; n.d. not determined. Phenotype baseline/under treatment: systolic blood pressure, plasma volume and plasma renin concentration in these models/genotypes have been determined in previous studies. For details see Table 2.

Group	Genotype	UNx	High salt diet	DOCA	Phenotype baseline	Phenotype under treatment
1	WT	sham			no	no
	WT	✓			no	no
2	Podo GC-A KO	✓	✓	✓	no	✓
3	WT	✓	✓	✓	no	✓
4	Podo GC-A KO	✓	✓		no	✓
5	GC-A KO	✓			✓	n.d.

Experimental group 1, UNx in wildtype mice (Tables 1, 2): As a proof of principle, the effect of EMPA on glomerular non-diabetic hyperfiltration in kidneys of uninephrectomized (UNx) wildtype (WT) animals was tested. WT animals received only UNx or sham surgery without any further intervention and were sacrificed 2 weeks post UNx. WT animals with UNx do not develop albuminuria and renal damage within several weeks post UNx (Staffel et al., 2017), why this model was not further monitored for progression of kidney disease. Experimental group 2, UNx/DOCA/salt in Podo GC-A KO (Tables 1, 2): The cardiac natriuretic peptides ANP and BNP activate the same receptor, the membrane-bound guanylyl cyclase-A (GC-A). GC-A is expressed in various cell types in the body and is critically involved in the regulation of the blood pressure and the extracellular volume. We have recently shown that podocytes have a strikingly high expression of GC-A (Staffel et al., 2017). Podocyte-specific deletion of GC-A (Podo GC-A KO) does not alter blood pressure, plasma volume and plasma renin concentration under control conditions (Tables 1, 2). Treatment of Podo GC-A KO with UNx/DOCA/salt not only resulted in arterial hypertension, hypervolemia and massive suppression of plasma renin concentration (Table 2, data from Staffel et al., 2017), but also in massive glomerular damage and albuminuria (Staffel et al., 2017). Since glomerular damage induced by UNx/DOCA/salt was markedly aggravated in Podo GC-A KO compared with Podo GC-A WT despite of similar effects on blood pressure, plasma volume and plasma renin concentration in both genotypes, podocytes of Podo GC-A KO mice appear to be more susceptible to these stressors. Therefore, this genetic model was used to induce severe glomerular damage. Podo GC-A KO mice received UNx and a 60 days-release pellet (150 mg) of the mineralocorticoid deoxycorticosterone acetate (DOCA) was implanted subcutaneously (Innovative Research of America, Sarasota, FL, United States). From day seven post UNx (regeneration from surgery), the mice were fed a high-salt diet (4% NaCl, ssniff-Spezialdiäten GmbH, Soest, Germany) until the end of the experiment. Due to severe kidney damage with marked albuminuria at 4 weeks post UNx the experiment was terminated prematurely. Experimental group 3, UNx/DOCA/salt in WT mice (Tables 1, 2): In order to generate a less aggressive kidney injury model with moderate albuminuria the described UNx/DOCA/salt model (group 2) was applied to WT C57BL/6J animals with only minor changes. As shown in Table 2 UNx/DOCA/salt in wildtype mice induces arterial hypertension, hypervolemia and a massive suppression in plasma renin concentration. A 21 days-release DOCA-pellet (50 mg) was implanted in WT animals at day seven post UNx. The high-salt diet started on the same day and mice were sacrificed after 4 weeks.

**TABLE 2:** Phenotype of kidney disease mouse models used in this study. UNx, unilateral nephrectomy; DOCA, deoxycorticosterone acetate; bw, body weight; n.d. not determined. Blood pressure, plasma volume and plasma renin concentration have been determined in these models by our group previously.

Group	Genotype	Arterial hypertension	Increase in plasma volume (in % of bw)	Plasma renin concentration	Glomerular damage
1	WT sham	no	no	n.d.	—
	WT UNx	no	no	n.d.	—
2	Podo GC-A KO UNx/DOCA/salt	+10 mmHg vs. baseline* ^a^	+0.38% vs. baseline* ^b^	2.9% of baseline* ^a^	Massive
3	WT UNx/DOCA/salt	+11 mmHg vs. baseline* ^a^	+0.40% vs. baseline* ^b^	3.1% of baseline* ^a^	Moderate
4	Podo GC-A KO UNx/salt	no^c^	no^c^	58.5% of baseline* ^c^	Mild
5	GC-A KO UNx	+17 mmHg vs. WT* ^d^	+0.29% vs. WT* ^d^	59.1% vs. WT* ^d^	Moderate
		Sham vs. UNx n.d.	Sham vs. UNx n.d.	Sham vs. UNx n.d.	

*p < 0.05 vs. baseline or WT, as indicated.

a Published in Staffel et al., 2017.

b Same mice as in Staffel et al., 2017, unpublished.

c Unpublished.

d Published in Demerath et al., 2014.

Experimental group 4, UNx/high salt in Podo GC-A KO (Tables 1, 2): As a model for mild glomerular damage/albuminuria uninephrectomized Podo GC-A KO mice were challenged with a high-salt diet (4% NaCl, ssniff-Spezialdiäten GmbH, Soest, Germany) for 6 weeks, but without concomitant DOCA treatment. As shown in Table 2, data of previous experiments showed that this model does not develop arterial hypertension or an increase in plasma volume, while plasma renin concentration was significantly suppressed.

Experimental group 5, UNx in GC-A KO (Tables 1, 2): In order to avoid the use of high-salt diet and DOCA, mice with global deletion of the receptor for ANP and BNP (GC-A KO) were chosen. Even under control conditions, GC-A KO mice are hypertensive (+17 mm Hg), hypervolemic (plasma volume + 0.3% of bodyweight) and have reduced plasma renin concentration (Lopez et al., 1995; Demerath et al., 2014). The effects of UNx on these parameters have not been determined in GC-A KO yet. To induce glomerular hyperfiltration, GC-A KO mice received UNx only and renal function was monitored for 6 weeks.

### EMPA Treatment and Urinary Glucose Measurement

EMPA treatment in all groups started 3 days before UNx at a dose of 30 mg/kg/day (Carbosynth Limited, Compton, United Kingdom) administered via drinking water. The EMPA concentration was adjusted to the drinking behavior of each experimental group, in particular for animals with increased water uptake during DOCA and high-salt diet. Fresh drinking water was prepared every 3–4 days. As proof of efficacy, urinary glucose levels were monitored on a regular base using a colorimetric glucose quantification kit (Cayman Chemical, Ann Arbor, MI, United States).

### GFR Measurement

GFR was assessed in conscious mice at prespecified time points post UNx using the transdermal GFR technology (MediBeacon Inc., Mannheim, Germany). Briefly, the back of the mice was shaved under 3% isoflurane anaesthesia and the GFR monitor was installed on the shaved skin. Background fluorescence signal was measured for 5 min before Fluorescein isothiocyanate (FITC)-labeled sinistrin (15 mg/100 g bodyweight) was injected intravenously. After injection, animals were placed in individual cages and the fluorescence signal in the skin was measured for 90 min in conscious and freely moving mice. The recorded clearance of FITC-sinistrin was used to calculate the excretion half-life t_1/2_ of FITC-sinistrin according to the manufacturer’s instructions (MPD Studio2 software; remove artifact filter; 3 compartment model with linear correction). FITC-sinstrin t_1/2_ was converted to GFR using a mouse-specific conversion factor (Schreiber et al., 2012): GFR [µl/min/100 g b. w.] = 14616, 8 [µl/100 g b. w.]/t_1/2_ [min]. Since we compare single kidney GFR in our analysis, GFR values of mice without UNx were divided by two.

### Urinary Albumin/Creatinine Measurement

Throughout the experiment, spot urine was collected each week to determine the albumin/creatinine ratio in the urine as a marker of progressing kidney damage. Albumin was quantified using a specific mouse albumin ELISA according to the manufacturer’s instructions (Dunn Labortechnik, Asbach, Germany). A colorimetric assay (improved Jaffe method) was used to determine creatinine (BioAssay Systems, Hayward, CA, United States).

### Metabolic Cages

For long-term assessment of drinking behavior and urine excretion, mice were kept in metabolic cages with free access to food and water for a period of 72 h. Urine was collected and drinking water was replaced every 24 h. The health status of animals was closely monitored by visual inspection and daily body weight measurements.

### Plasma Renin Concentration

At the end of the experiment, blood samples were taken from the facial vein of conscious mice and mice were killed by cervical dislocation thereafter. Determination of plasma renin concentration (PRC) in these plasma samples was based on the generation of angiotensin I after the addition of plasma from bilaterally nephrectomized male rats as excess renin substrate. The generated angiotensin I [ng/ml*h^−1^] was determined by ELISA [Angiotensin I (PRA) ELISA; IBL International, Germany].

### Masson-Goldner-Trichrome Staining

At the end of the experiment, mice were killed by cervical dislocation and prepared for perfusion via the abdominal aorta. Kidneys were rinsed with a 0.9% NaCl solution, perfusion-fixed with 4% paraformaldehyde (3 min; 100 mmHg constant pressure) and stored in 70% methanol for paraffin-embedding. After sectioning (5 µm), kidney sections were stained for collagenous, fibrotic tissue using a standard Masson-Goldner-Trichrome staining protocol (Sigma-Aldrich, HT15-1KT, Taufkirchen, Germany). Stained kidney sections were examined by light microscopy (Axio Observer 7, Carl Zeiss, Jena, Germany). For unbiased quantification of the fibrotic tissue areas (blue staining) we used the Zeiss ZEN Intellesis Image Segmentation software (Carl Zeiss, Jena, Germany). In brief, the software uses a machine-learning algorithm for automated identification of stained areas based on an individual training for each segmentation class. We defined three different classes recognizing background, healthy kidney or fibrotic tissue areas and used the fibrosis/kidney ratio as read out parameter for our analysis.

### EMPA Plasma Concentration

Blood was collected from WT animals after a 2 weeks EMPA treatment (30 mg/kg/day) via submandibular puncture of the facial vein. Total and free EMPA concentration in plasma were determined by HPLC-MS/MS. Further details regarding sample preparation and HPLC-MS/MS measurement are given in the supplementary data.

### Plasma Volume Measurement

Plasma volume was measured in conscious mice by single intravenous injection of 50 µl FITC-labeled bovine serum albumin (BSA; Sigma-Aldrich, Taufkirchen, Germany) under mild isoflurane anesthesia. A 10 µl blood sample was collected from the tail vein before and 20 min post FITC-BSA injection. Plasma fluorescence was measured on a Nanodrop 3,000 (Peqlab Biotechnologie GmbH, Erlangen, Germany) and FITC-BSA concentration was calculated according to an appropriate standard curve. We used the equation c (FITC-BSA stock)* v (FITC-BSA stock) = c (FITC-BSA plasma)* v (plasma) to determine the plasma volume of mice.

### Real-Time PCR

Renal mRNA was extracted from 4 paraffin-embedded kidney sections (15 µm) per sample using the Quick-RNA FFPE Miniprep-Kit (Zymo Research Europe GmbH, Freiburg, Germany). Cardiac mRNA was extracted from left ventricles of excised hearts using the RNeasy Mini Kit (Qiagen, Hilden, Germany) according to the manufacturer’s recommendation. cDNA was transcribed from 1 µg RNA using random primers, PCR nucleotide mix, RNasin ribonuclease inhibitor, reverse transcriptase and reverse transcriptase 5x reaction buffer (Promega GmbH, Walldorf, Germany) for 1 h at 37°C. mRNA abundance of renal (fibronectin, α-smooth muscle actin, collagen 1a1) and cardiac target genes (BNP, α-smooth muscle actin, TGF-β, collagen 1a1, collagen 3a1) were measured using the SYBR Green PCR (Roche Diagnostics Deutschland GmbH, Mannheim, Germany) or TaqMan Gene Expression (Thermo Fisher Scientific GmbH, Dreieich, Germany) detection method. For relative mRNA expression analysis according to the comparative threshold cycle (Ct) relative quantification analysis method (Livak and Schmittgen, 2001) Rpl-32 (kidney) and Gapdh (heart) were used as housekeeper genes. Relative target gene mRNA expression are shown as percentage of the expression level in H_2_O animals of the respective genotype (100%). Primer sequences and the corresponding detection methods can be found in Supplementary Tables S1, S2.

### Statistics

Data are shown as mean ± SEM. For single-group comparisons, an unpaired Student’s *t* test was used to calculate the level of significance. Accordingly, for multi-group comparisons at different time points, a two-way ANOVA followed by a Bonferroni post hoc test was used. All statistical analyses were performed using the GraphPad Prism software. Differences between groups were considered significant at a *p* < 0.05.

## Results

### Proof of Principle: Plasma EMPA Concentration and Increased Glucose Excretion in WT Animals

**FIGURE 1:**
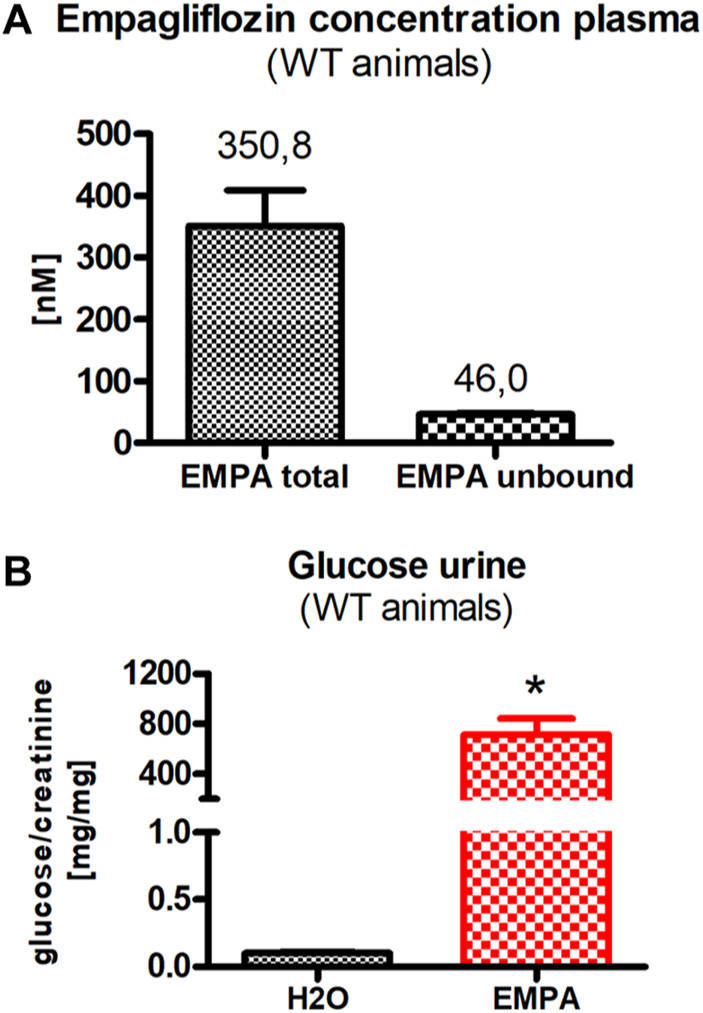
WT animals (*n* = 3 animals per group) were treated with EMPA (30 mg/kg/d) for 2 weeks and untreated animals were used as control. **(A)** Plasma concentration of protein-bound and unbound EMPA in EMPA-treated animals. **(B)** Urine glucose concentration, normalized to creatinine concentration (mg glucose/mg creatinine), was highly increased in EMPA-treated animals compared with H_2_O control animals. Bar charts show mean values (±SEM) and asterisks indicate *p* < 0.05. EMPA, empagliflozin; WT, wildtype.

To verify that EMPA administration via the drinking water results in therapeutic plasma levels, EMPA plasma concentration of WT animals was determined by HPLC-MS/MS. After 2 weeks of EMPA treatment, total plasma EMPA concentration was 350.8 nM, which corresponds to the plasma concentration determined in clinical trials (Scheen, 2014) (Figure 1A). Protein precipitation revealed that 87% of EMPA was bound to plasma proteins and the concentration of unbound EMPA was 46 nM. In line with the expected inhibition of SGLT2, urinary glucose/creatinine ratio was markedly increased in EMPA treated WT animals (714.31 ± 126.15 mg/mg; Figure 1B) compared with H_2_O control animals (0.1 ± 0.011 mg/mg; Figure 1B).

### EMPA Reduces Non-Diabetic Hyperfiltration in WT Animals

**FIGURE 2:**
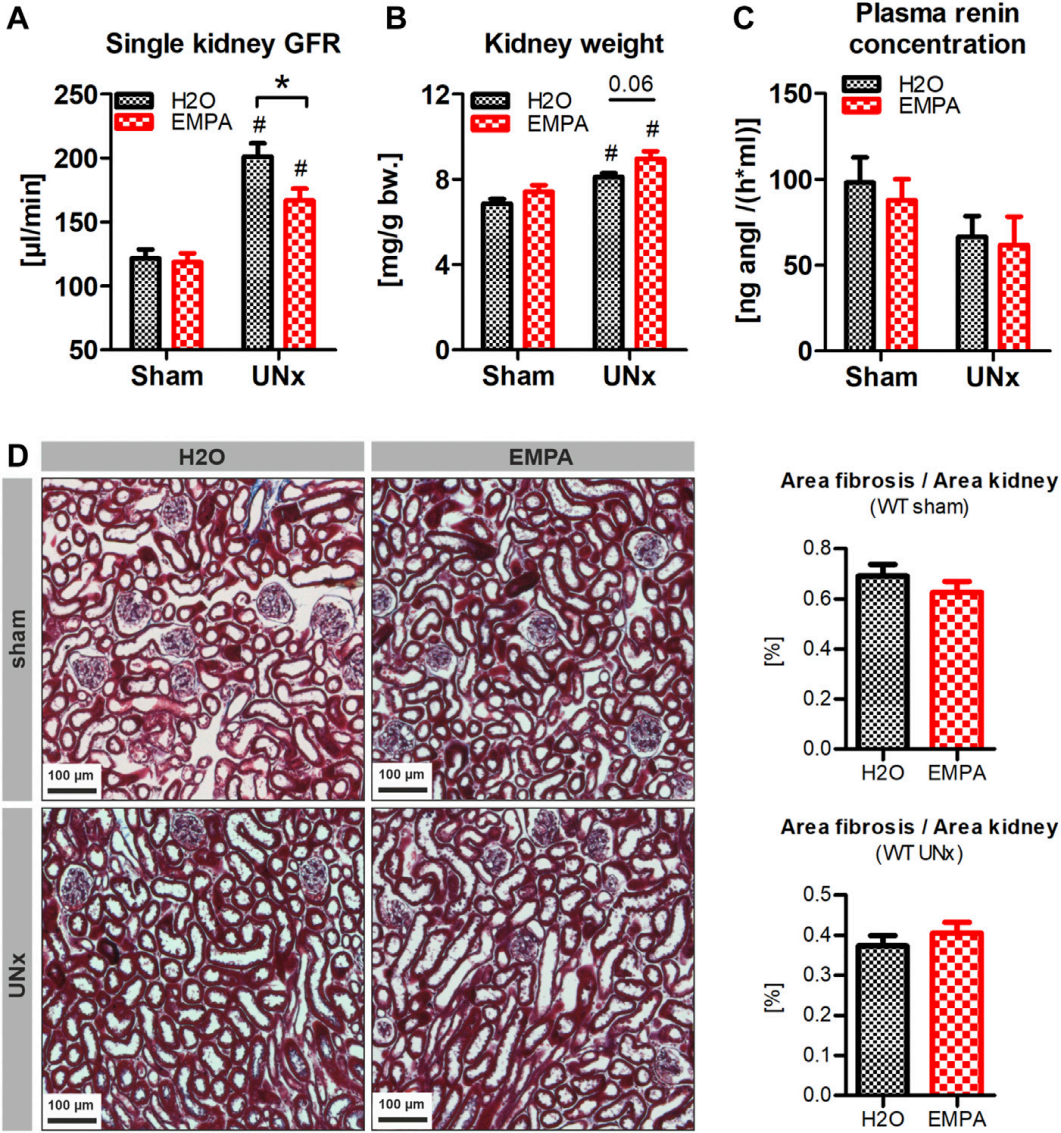
Reduction of UNx-induced hyperfiltration in EMPA-treated mice. WT animals (*n* = 7–8 animals per group) received UNx or sham surgery and were treated with EMPA (30 mg/kg/d, drinking water) for 2 weeks. **(A)** EMPA had no effect on single kidney GFR in sham animals (GFR values were divided by two) but significantly reduced hyperfiltration in uninephrectomized animals. **(B)** Kidney weights are illustrated in relation to bodyweight before treatment or surgery. **(C)** Plasma renin concentration 2 weeks after UNx. **(D)** Representative images of kidney histology and fibrosis (blue, Masson-Goldner staining) and fibrosis/kidney area ratio, measured by automated signal quantification. Bar charts show mean values (±SEM) and asterisks indicate *p* < 0.05 comparing groups connected by squared brackets; # *p* < 0.05 vs. sham. EMPA, empagliflozin; WT, wildtype; GFR, glomerular filtration rate; UNx, unilateral nephrectomy; bw, bodyweight.

Here, we investigated whether EMPA affects GFR of control (sham-operated) and hyperfiltrating (UNx) kidneys of non-diabetic WT animals. To induce hyperfiltration, the left kidney of WT animals was excised and single kidney GFR was determined 2 weeks later. EMPA had no effect on GFR in sham-operated animals (Figure 2A). UNx induced a marked increase in single kidney GFR compared to sham-operated control animals. This effect was blunted in EMPA treated animals, in which hyperfiltration in response to UNx was significantly reduced compared with H_2_O treated UNx animals (65 vs. 41% GFR increase, Figure 2A). UNx not only results in an increase in GFR but also in kidney hypertrophy. This parallelism of GFR and kidney weight was abolished in EMPA-treated mice, because EMPA reduced GFR in UNx mice, but kidney weight tended to be even slightly higher in EMPA than in H_2_0 mice (*p* = 0.06; Figure 2B). Plasma renin concentration was not altered by EMPA in sham or UNx mice (Figure 2C). Histological examination of renal slices did not reveal any signs of enhanced fibrosis in response to UNx and EMPA did not have any effect on kidney histology and fibrosis (Figure 2D).

GFR and progression of renal damage were not further monitored in these mice, as previous studies proved that WT animals with UNx but without any further intervention do not even develop albuminuria and renal damage within several weeks post UNx (Staffel et al., 2017). Therefore, this model is unsuitable for investigations of nephroprotective mechanisms of EMPA and UNx was combined with additional treatments and genetic alterations to enhance glomerular damage.

### Hyperfiltration Model With Severe Albuminuria: EMPA Effect on Podo GC-A KO Mice With UNx/DOCA/Salt Treatment

**FIGURE 3:**
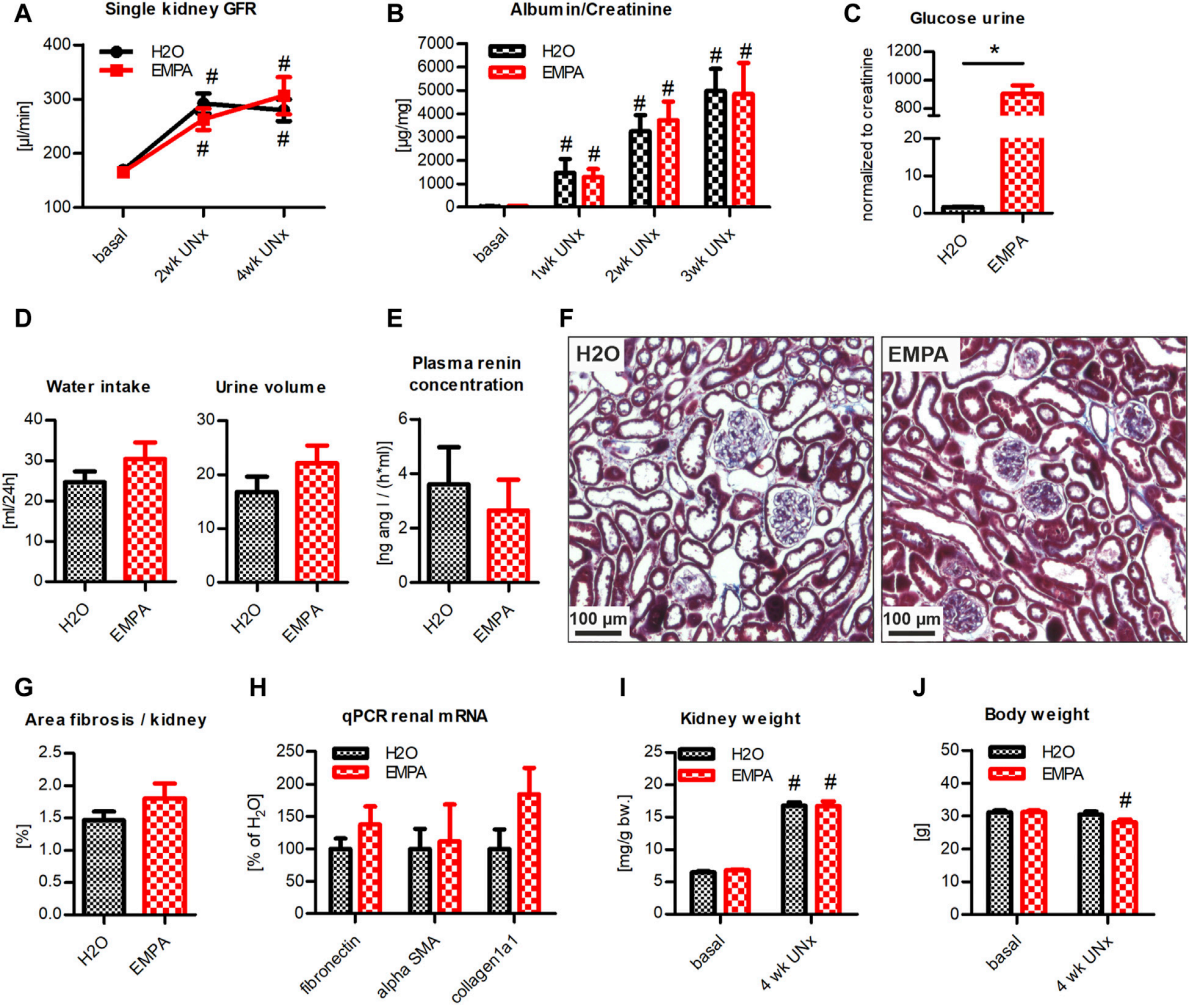
EMPA does not protect from renal damage in Podo GC-A KO mice with UNx/DOCA/salt. Mice with podocyte-specific deletion of the natriuretic peptide receptor GC-A (Podo GC-A KO) were subjected to UNx and treated with DOCA and 4% high NaCl diet. 3 days prior to UNx animals were split in 2 groups (*n* = 9–10 animals per group), an EMPA-treated cohort and a H_2_O control cohort. **(A)** Single kidney GFR was increased at 2 and 4 weeks post UNx compared to baseline GFR (baseline GFR values were divided by two) with no differences between groups. **(B)** DOCA/salt treatment induced massive albuminuria in all mice independent from treatment with EMPA. **(C)** EMPA increased urine glucose concentration in spot urine 2 weeks post UNx (normalized to creatinine). **(D)** Drinking behavior and urine volume of mice in metabolic cages (ml/24 h). **(E)** Plasma renin concentration. **(F)** Kidney histology and fibrosis (blue) was examined using standard Masson-Goldner staining. Representative images revealed fibrotic collagen deposits in H_2_O and EMPA treated animals with no significant difference in fibrosis/kidney area ratio **(G)** between groups, measured by automated signal quantification. **(H)** EMPA treatment did not significantly alter mRNA expression of fibronectin, alpha-SMA and collagen1a1. **(I)** Kidney weight (related to body weight at baseline) was not affected by EMPA treatment, while bodyweight itself was reduced in EMPA treated DOCA/salt mice **(J)**. Bar charts show mean values (±SEM). * *p* < 0.05 EMPA vs. H_2_0. # *p* < 0.05 vs. basal condition. EMPA, empagliflozin; GFR, glomerular filtration rate; UNx (U), unilateral nephrectomy; wk, weeks; bw, bodyweight.

Podo GC-A KO mice with UNx/DOCA/salt treatment were used to study the nephroprotective potential of EMPA in a model system for severe glomerular damage and progressive renal failure. After 2 weeks of UNx/DOCA/salt treatment, single kidney GFR increased in Podo GC-A KO mice to +172% in the H_2_O group and +158% in the EMPA group compared to baseline levels (Figure 3A). The slight trend toward reduced hyperfiltration in EMPA treated animals did not reach statistical significance and did not persist until week 4. The induced damage of the glomerular filter resulted in a massive increase in albuminuria (∼150x compared to baseline) to the same extent in H_2_O and EMPA treated animals (Figure 3B). Due to incipient symptoms of nephrotic syndrome in individual animals of both groups, the experiment was terminated prematurely after 4 weeks. EMPA induced glucosuria (Figure 3C) to a similar extent as in untreated wildtype mice (Figure 1B). Preexisting diuresis and increased water intake, caused by DOCA/salt treatment, were numerically higher in EMPA than in H_2_O mice (+5.4 and +5.7 ml respectively). However, this numerical difference did not reach statistical significance (Figure 3D). Plasma renin concentration was massively suppressed compared with untreated wildtype mice (shown in Figure 2) and EMPA did not significantly alter plasma renin concentration (Figure 3E). Histological examination of kidney sections revealed no differences in glomerular or tubular morphology between H_2_O and EMPA treated animals. Both groups showed the characteristic model-dependent collagen casts and slight glomerular hypertrophy (Figure 3F) (Staffel et al., 2017). Increased collagenous fibrotic structures, visualized by Masson-Goldner staining, were found in glomeruli of all animals (compared to healthy kidneys; for comparison see Figure 2), and automated quantification of fibrotic tissue in whole kidney overview images confirmed no significant effect of EMPA treatment on renal fibrosis (Figure 3G). In line with the histological examination, qPCR did not reveal significant differences in renal mRNA expression of the fibrosis genes fibronectin, alpha-SMA and collagen1a1 between the H_2_O and EMPA groups (Figure 3H). Likewise, marker genes for cardiac fibrosis were not altered by EMPA treatment (Supplementary Figure S1). In response to the removal of the left kidney and subsequent hyperfiltration, all animals developed pronounced hypertrophy of the remaining right kidney irrespective of the treatment (Figure 3I). Body weight did not change in the H_2_0 group but decreased significantly in the EMPA group during 4 weeks of DOCA/salt treatment (−9.8% vs. baseline, Figure 3J). Overall, the progression of renal damage caused by a combination of a genetic model with increased glomerular vulnerability (Podo GC-A KO) and the UNx/DOCA/salt model was not ameliorated by EMPA treatment.

### Hyperfiltration Model With Moderate Albuminuria: EMPA Effect on WT Animals With UNx/DOCA/Salt Treatment

**FIGURE 4:**
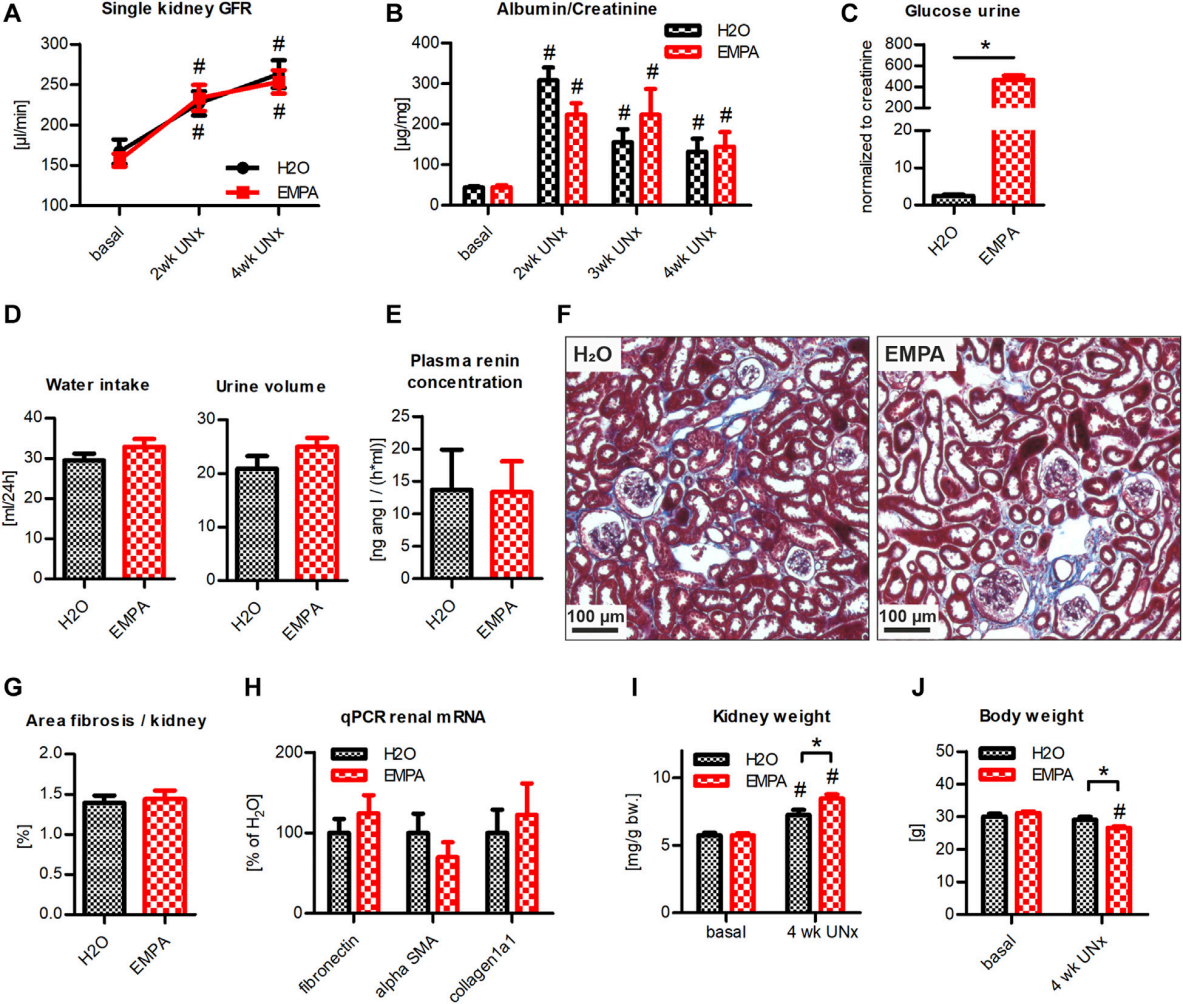
EMPA is not renoprotective in WT mice with UNx/DOCA/salt. WT animals were subjected to UNx and DOCA/high salt. 3 days prior to UNx animals were split in 2 groups (*n* = 7–9 animals per group), treated either with EMPA or H_2_O (control). **(A)** Hyperfiltration developed within 4 weeks post UNx without any differences between H_2_O and EMPA treated mice. **(B)** Albumin excretion increased after DOCA/salt treatment in both groups and EMPA-treated animals showed substantial urinary glucose loss **(C)**. **(D)** Water uptake and urine output per day. **(E)** Determination of plasma renin concentration. No differences in tubulointerstitial fibrosis were observed by visual inspection **(F)** or fibrosis quantification **(G)** in kidney sections of H_2_O and EMPA animals. **(H)** Determination of mRNA abundance of fibronectin, alpha-SMA and collagen1a1 by qPCR. **(I)** EMPA increased kidney weight after 4 weeks of DOCA/salt treatment. Kidney weight was related to bodyweight at baseline. **(J)** Body weight was reduced in EMPA treated mice. Bar charts show mean values (±SEM). * *p* < 0.05 EMPA vs. H_2_0. # *p* < 0.05 vs. basal condition. EMPA, empagliflozin; GFR, glomerular filtration rate; UNx (U), unilateral nephrectomy; wk, weeks; bw, bodyweight.

Since a potential nephroprotective effect of EMPA might be masked in an aggressive kidney injury model, we repeated the UNx/DOCA/salt model in a cohort of WT animals. Within the first 2 weeks post UNx, single kidney GFR rose to 135% in control vs. 149% in EMPA treated animals compared to baseline GFR levels (Figure 4A). At 4 weeks, hyperfiltration had further increased to 157 and 161% (Figure 4A), respectively, with no significant differences between groups. Animals of both groups developed a persistent moderate albuminuria throughout the course of the experiment (Figure 4B). Again, inhibition of SGLT2 by EMPA caused a massive loss of glucose in the urine (Figure 4C). DOCA-high salt diet induced marked diuresis and water uptake (Figure 4D). There was a slight trend toward increased water intake and urine volume in the EMPA group, without reaching statistical significance (Figure 4D). Plasma renin concentration was markedly reduced compared to untreated WT mice (shown in Figure 2) and was not affected by EMPA treatment (Figure 4E). In line with glomerular damage, both groups developed renal fibrosis to the same extent, visualized by histological staining of collagen deposits in glomeruli and tubulointerstitial areas (Figures 4F,G). Similarly, renal mRNA abundance of fibronectin, alpha-SMA and collagen1a1 was not different between H_2_O and EMPA treated mice (Figure 4H) and cardiac fibrosis genes were unaffected by EMPA treatment (Supplementary Figure S1). While the extent of hyperfiltration was similar in both groups (Figure 4A), the increase in kidney weight was significantly greater in EMPA treated animals (kidney weight was related to body weight at baseline, Figure 4I). Body weight remained stable in the H_2_O group, while it decreased significantly in the EMPA group (Figure 4J). In conclusion, no protective effect of EMPA on hyperfiltration, albuminuria or renal fibrosis was detected in this model.

### Hyperfiltration Model With Mild Albuminuria: EMPA Effect on Podo GC-A KO Mice With UNx/Salt Treatment

**FIGURE 5:**
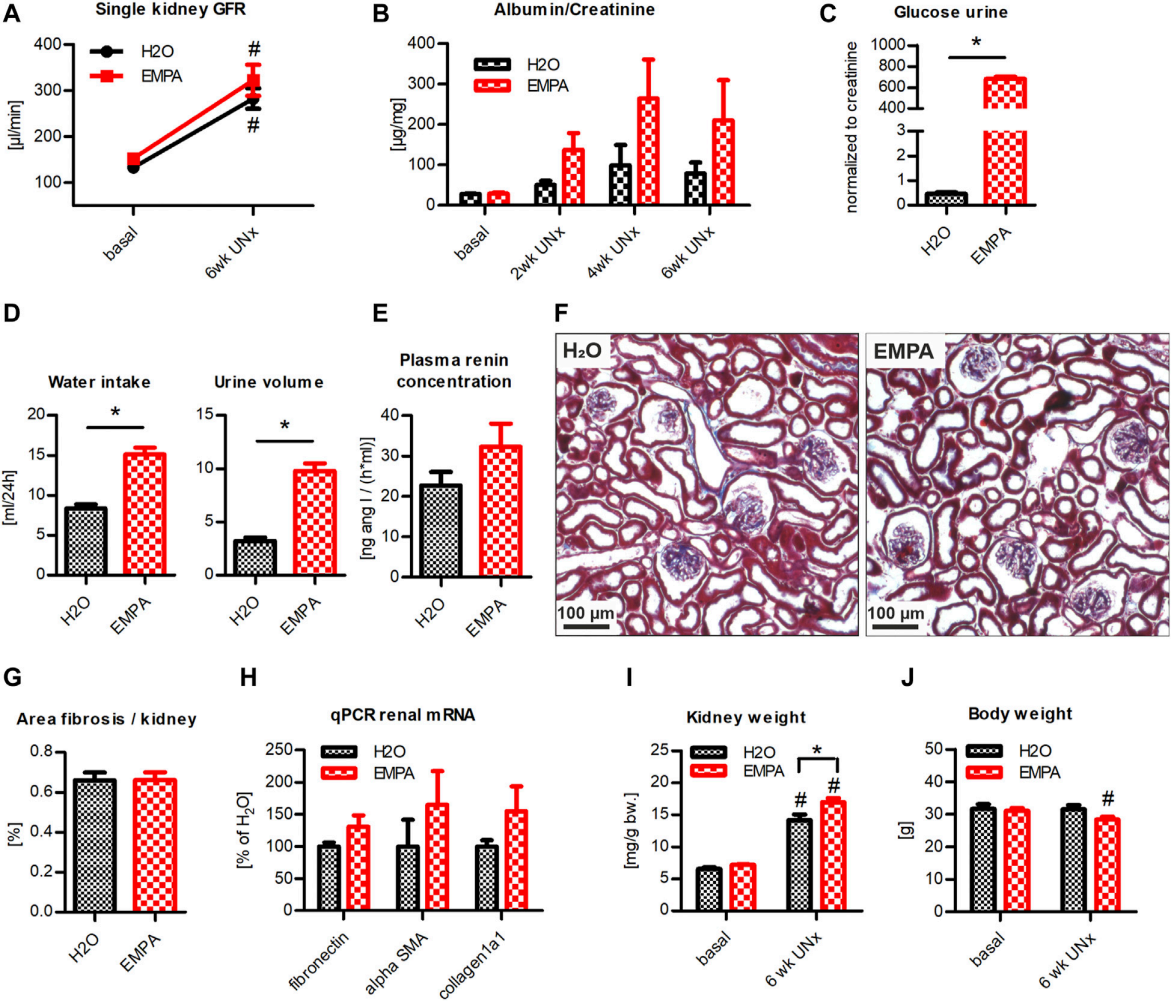
EMPA does not protect from renal damage in Podo GC-A KO mice with UNx/salt. Mice with podocyte-specific deletion of GC-A (Podo GC-A KO) were subjected to UNx and treated with 4% high NaCl diet for 6 weeks. 3 days prior to UNx animals were split in 2 groups (*n* = 7–8 animals per group), an EMPA-treated cohort and a H_2_O control cohort. **(A)** Measurement of single kidney GFR before (baseline GFR values were divided by two) and 6 weeks post UNx/high salt treatment with no effect of EMPA. **(B)** Urine albumin/creatinine ratio tended to be elevated in EMPA mice, without statistical significance. EMPA-induced glucosuria **(C)**, increased water uptake and diuresis **(D)**. **(E)** Plasma renin concentration was not altered by EMPA. **(F)** Fibrotic tissue was stained in kidney-sections using Masson-Goldner-staining, but no differences in fibrotic area (**G**; normalized to whole kidney tissue) could be detected between groups. Likewise no significant differences in fibronectin, alpha-SMA and collagen1a1 between EMPA and H2O groups were detectable **(H)**. **(I)** UNx/salt treatment for 6 weeks induced marked renal hypertrophy in both H_2_O and EMPA treated mice. This hypertrophy response was further augmented by EMPA. **(J)** Body weight remained stable in H_2_O treated mice but decreased under EMPA treatment. Bar charts show mean values (±SEM). * *p* < 0.05 EMPA vs. H_2_0. # *p* < 0.05 vs. basal condition. EMPA, empagliflozin; GFR, glomerular filtration rate; wk, weeks; bw, bodyweight.

In a next approach, we examined the effect of EMPA on a rather mild kidney damage model. Therefore, Podo GCA KO mice received UNx in combination with a high salt diet, but without concomitant DOCA treatment. Expecting a slow progression of renal damage, the experimental protocol was extended from 4 to 6 weeks. At 6 weeks post UNx, single kidney GFR was more than doubled in both groups (212 vs. 210% compared to basal levels) with no effect of EMPA on either healthy (basal; Figure 5A) or hyperfiltrating kidneys (6 weeks; Figure 5A). Glomerular damage was rather mild, as indicated by low albumin levels in the urine of control animals (Figure 5B). Urinary albumin excretion was numerically slightly increased in EMPA-treated animals, but the difference did not reach statistical significance. EMPA induced glucosuria to a similar extent as in the other models (Figure 5C). Water intake and diuresis were markedly lower compared with the DOCA/salt treated mice shown in Figures 3, 4 and EMPA induced significant increases in both parameters (Figure 5D). Plasma renin concentration was not altered by EMPA (Figure 5E). Histological (Figure 5F) and quantitative (Figure 5G) examination of fibrotic collagen deposits in kidney sections revealed no difference between groups and results were similar to those obtained for healthy kidneys (for comparison see Figure 2). Fibronectin, alpha-SMA and collagen1a1 mRNA levels tended to be higher in EMPA treated mice, without statistically significant difference (Figure 5H). Matching the strong increase in single kidney GFR, all animals, independent of treatment, showed a massive renal hypertrophy (>2x increase in kidney weight compared to control kidneys) after UNx. This effect was pronounced in EMPA treated mice, since kidney weight (related to body weight at baseline) was significantly higher than in H_2_0 treated mice (Figure 5I). As in the models before (Figures 3–5) the body weights of EMPA treated mice decreased in the course of the treatment phase while it remained constant in the H_2_O group (Figure 5J).

### Hyperfiltration Model With Genetic Arterial Hypertension and Hypervolemia: EMPA Effect on Global GC-A KO Mice With UNx

**FIGURE 6:**
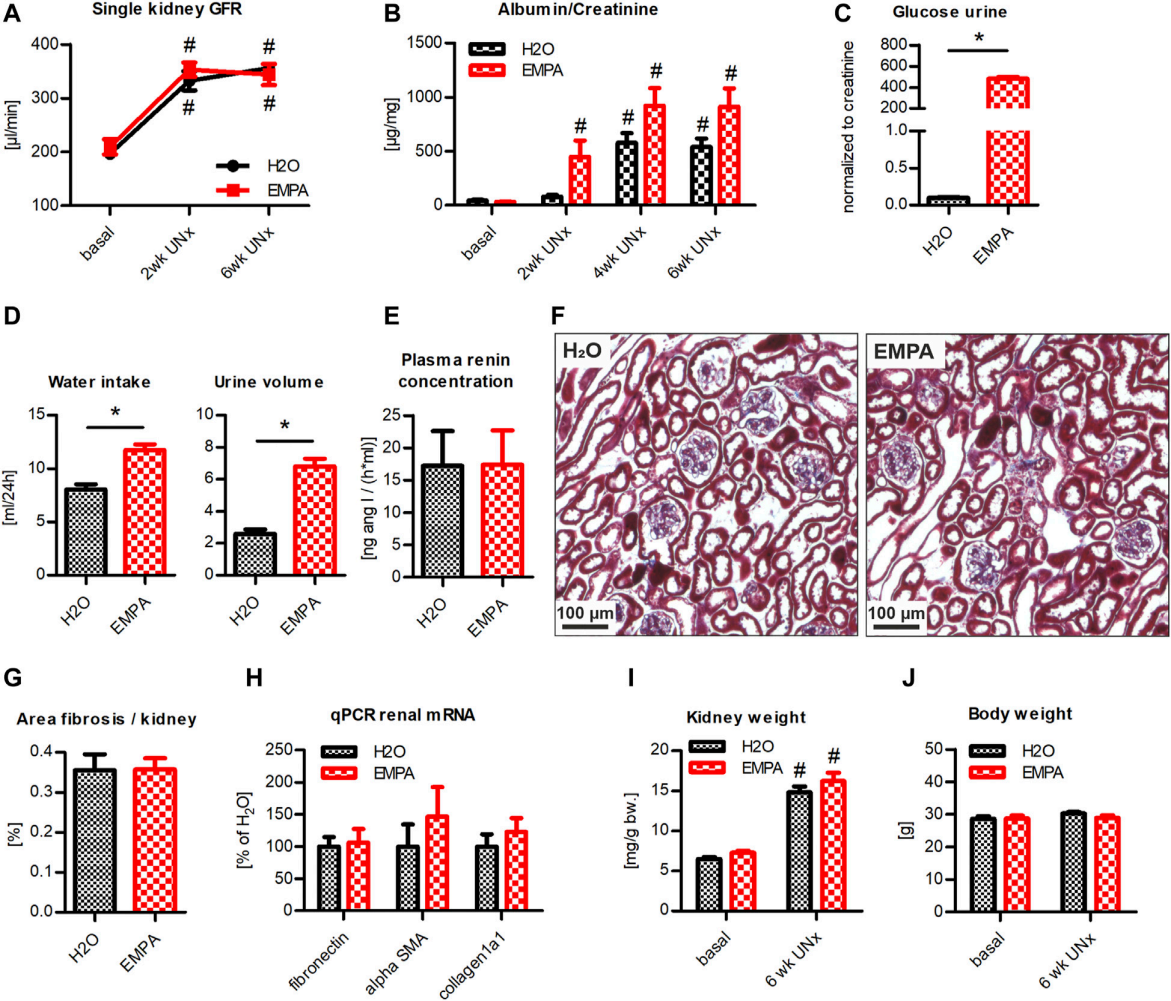
EMPA does not protect from renal damage in uninephrectomized GC-A KO mice. Mice with deletion of the GC-A (GC-A KO) were subjected to UNx and split in 2 groups (*n* = 8–9 animals per group), an EMPA-treated cohort and a H_2_O control cohort. **(A)** Single kidney GFR was elevated at 2 and 6 weeks post UNx in both groups. EMPA did not affect glomerular hyperfiltration. **(B)** Urine albumin excretion increased in all animals with a tendency towards higher albuminuria in EMPA-treated animals. SGLT2 inhibition caused glucosuria **(C)**, increased water intake and diuresis **(D)**, but no changes in plasma renin concentration **(E)**. **(F)** Masson-Goldner staining on kidney sections showed almost no fibrotic areas in all animals regardless of EMPA-treatment. This was confirmed by automated quantitative image analysis **(G)**. **(H)** Fibrosis genes fibronectin, alpha-SMA and collagen1a1 were not affected by EMPA. **(I)** Kidney/bodyweight and bodyweight **(J)** were not significantly different between EMPA and H_2_O mice. Bar charts show mean values (±SEM). * *p* < 0.05 vs. H_2_O; # *p* < 0.05 vs. baseline. EMPA, empagliflozin; GFR, glomerular filtration rate; UNx, unilateral nephrectomy; wk, weeks; bw, bodyweight.

As a final approach, GC-A knockout mice, which are hypervolemic and hypertensive without DOCA/salt treatment (Tables 1, 2) were subjected to UNx. UNx in GC-A KO mice induced a constant hyperfiltration in the remaining kidney that was not affected by EMPA treatment (H_2_O: 170–180% vs. EMPA: 165–170% compared to basal levels) (Figure 6A). Albuminuria increased in both groups within the first 4 weeks post UNx until it reached a stable level (Figure 6B). A strong trend towards higher urinary albumin/creatinine ratio in EMPA treated mice compared with H_2_0 control animals was detected, but the observed differences did not reach statistical significance (Figure 6B). EMPA induced urinary glucose loss (Figure 6C), marked diuresis (+4.21 ml/24 h; Figure 6D) and increased water uptake (Figure 6D). EMPA did not affect the plasma volume (H_2_O: 1.20 ± 0.22 ml; EMPA: 1.19 ± 0.26 ml, data not shown), which has been shown to be elevated in GC-A KO animals under control conditions previously (Sabrane et al., 2005; Demerath et al., 2014). As in the other models, EMPA did not affect plasma renin concentration (Figure 6E). Visual inspection of kidney sections with Masson-Goldner staining revealed no clear signs for renal fibrosis in H_2_O and EMPA-treated GC-A KO animals (Figure 6F). These results were confirmed by an unbiased automated quantification analysis (Figure 6G) and by qPCR (Figure 6H). Moreover, EMPA did not affect cardiac fibrosis markers (Supplementary Figure S1). Again, we observed renal hypertrophy after UNx, but in this model, EMPA had no significant effects on elevated kidney weights (Figure 6I) or body weight (Figure 6J).

In summary, SGLT2 inhibition by EMPA reduced single kidney GFR in hyperfiltrating kidneys of WT mice independent of the glycemic state of the animal. Moreover, EMPA treatment increased glucosuria in all models with varying degrees of renal dysfunction. However, prominent markers of kidney damage such as albuminuria and renal fibrosis were not ameliorated by EMPA treatment, probably due to the lack of an effect of EMPA on single kidney GFR under challenging conditions like high salt intake, hypervolemia or arterial hypertension.

## Discussion

The nephroprotective potential of SGLT2 inhibitors canagliflozin, dapagliflozin and empagliflozin has been impressively demonstrated in several clinical trials in patients with type II diabetes (Zinman et al., 2015; Wanner et al., 2016; Neal et al., 2017; Wiviott et al., 2019). Furthermore, it is now clear from the DAPA-CKD trial in non-diabetic patients with CKD that the therapeutic impact of SGLT2 inhibitors by far exceeds the expectations for a “classical” antidiabetic drug. In the latter trial, dapagliflozin improved renal function, indicated by stabilization of GFR, reduced risk of end stage renal disease and death from renal failure in 1,398 patients with non-diabetic CKD, primarily caused by ischemic/hypertensive nephropathy, immunoglobulin A nephropathy and focal segmental glomerulo-sclerosis (Heerspink et al., 2020). Notably, the DAPA-CKD study was terminated early due to overwhelming efficacy and renal beneficial effects were observed in patients with different stages of CKD, independent from baseline GFR or urinary albumin levels. Given this overwhelming evidence, it is surprising that data from preclinical studies in rodents with non-diabetic renal damage, which are inevitable to identify the mechanistic background behind nephroprotection, have so far provided rather inconsistent results. A number of *in vivo* studies reported no improvement of renal impairment by SGLT2 inhibitors caused by, e.g., 5/6 nephrectomy in rats (Zhang et al., 2016; Rajasekeran et al., 2018), polycystic kidney disease in rats (Kapoor et al., 2015), oxalate-induced nephrocalcinosis in mice (Ma et al., 2017) and adenine-induced fibrosis in rats (Yamazaki et al., 2020). On the contrary, data supporting a renal benefit by SGLT2 inhibition were provided from disease models like ischemia-reperfusion injury (Chang et al., 2016), unilateral ureteric obstruction (Abbas et al., 2018), protein-overload proteinuria (Cassis et al., 2018), Ang II-dependent hypertension (Castoldi et al., 2020), cyclosporine-A nephropathy (Castoldi et al., 2021), adenine nephropathy (Yamato et al., 2020) and salt-sensitive hypertension in uninephrectomized rats (Kim et al., 2019). It is worth mentioning that the discrepant results in the aforementioned studies are not due to the different SGLT2 inhibitors used, as both positive and negative results were reported for most of the compounds.

This study investigated the renoprotective effect of the SGLT2 inhibitor empagliflozin in 4 different mouse models with varying degrees of non-diabetic hyperfiltration and albuminuria. The administration of empagliflozin via the drinking water at a dose of 30 mg/kg per day resulted in a total plasma concentration of 350 nM, which corresponds to the plasma concentration achieved in clinical trials (Scheen, 2014). The plasma concentration of the unbound empagliflozin was 46 nM, an ideal concentration to selectively inhibit SGLT2 (IC50: 3.1 nM) in the early proximal tubule, but not SGLT1 (IC50: 8,300 nM) in late proximal tubule S3 segments (Grempler et al., 2012). It has to be mentioned that the determination of empagliflozin plasma concentration was performed in otherwise untreated wildtype mice and water intake in these mice is markedly lower than in some of the models used in this study, especially the DOCA models. In order to avoid overdosing of empagliflozin as a result of elevated daily water intake, the empagliflozin concentration in the drinking water was adjusted to the water intake of the respective models. Moreover, a plasma concentration of 46 nM unbound EMPA should be high enough to guarantee a permanent inhibition of SGLT2 even considering possible variations in drinking behavior, i.e., reduced water intake in individual animals. On the other hand, potential overdosing of EMPA could result in an additional blockade of SGLT1, which reabsorbs parts of the filtered glucose from late proximal tubules. Besides the direct effects on renal glucose excretion and glucose absorption in the small intestine, where it is highly expressed, SGLT1 inhibition would also block intrarenal signaling pathways. Thus, while the localization of SGLT2 in the early parts of the proximal tubule and of SGLT1 in the later parts of the proximal tubule are well established, SGLT1 has recently also been shown to be expressed in the luminal membrane of the thick ascending limb and the macula densa (Madunić et al., 2017; Zhang et al., 2019), where it modulates the TGF mechanism via regulation of the NO synthase NOS1 (Song et al., 2019). Moreover, since blockade of SGLT1 itself has a nephroprotective effect, overdose of SGLT2 inhibitor could in principle act via this mechanism (Nespoux et al., 2019). However, since the concentration of unbound EMPA is 180-fold below the IC50 of SGLT1 it is not expected to significantly inhibit SGLT1 even in possible cases of excessive water intake and associated overdosing.

In accordance with the tubular hypothesis of activation of the TGF system (Vallon and Thomson, 2020), data from this study demonstrate for the first time in mice the GFR lowering effect of SGLT2 inhibition in hyperfiltrating (UNx), non-diabetic kidneys, whereas EMPA had no effect on GFR of normofiltrating (sham), healthy kidneys. Recent studies in rats (Thomson and Vallon, 2021) and humans (van Bommel et al., 2020) showed evidence that the reduction of GFR relies on declining glomerular capillary pressure probably by TGF-mediated preglomerular vasoconstriction as well as postglomerular vasorelaxation, at least under diabetic conditions. Surprisingly, the GFR reducing effect of EMPA was totally abolished in our disease models, regardless of the severity of hyperfiltration-induced glomerular damage. Development of albuminuria and tubulointerstitial fibrosis was not prevented by EMPA in our experimental cohorts, unlike results obtained from uninephrectomized rats with high salt-induced hypertension (Kim et al., 2019). Possible explanations are so far only hypothetical and cannot explain the species differences, but the induced extracellular volume expansion in our disease models either by the use of high-salt diet (±DOCA) or deletion of the GC-A receptor (Demerath et al., 2014) might regulate the sensitivity of the tubuloglomerular feedback mechanism (TGF), for instance by inhibition of the renin-angiotensin-system (RAS). Cells of the macula densa and vascular smooth muscle cells express the AT1 receptor and it is known from microperfusion studies that angiotensin II, either applied systemically or via peritubular infusion, enhances the TGF-mediated vasoconstriction at the afferent arteriole of the glomerulus (Mitchell and Navar, 1988; Wang et al., 2001). Conversely, pharmacological blockade of angiotensin II formation by ACE inhibitors or angiotensin II signaling by AT1 receptor blockers markedly attenuated the TGF response (Schnermann, 2015). Complete genetic deletion of AT1 receptors or ACE completely abolished the TGF response to increases in NaCl load at the macula densa, further underlining the critical role of angiotensin II (Schnermann et al., 1997; Traynor et al., 1999). Due to different genetic backgrounds of the mouse strains used in this study, a direct comparison of plasma renin concentration between the groups is not possible in all cases. However, our data, together with results of previous studies (Demerath et al., 2014; Staffel et al., 2017), clearly show that the circulating renin-angiotensin system is suppressed to different degrees in all disease models and EMPA does not alter this suppression. Therefore, low angiotensin II levels can be expected in our experimental models and might result in an inactivated TGF system. Since an intact TGF response is needed for the EMPA-induced reduction of glomerular hyperfiltration, suppression of plasma renin concentration in our models might by responsible for the lack of an EMPA effect on hyperfiltration. However, a partial argument against this hypothesis is that the GFR-lowering effect of SGLT2 inhibitors also occurred in clinical trials with non-diabetic CKD patients receiving RAS blockers (Cherney et al., 2020; Heerspink et al., 2020). On the other hand, the extent to which global pharmacological inhibition of the RAS affects the angiotensin II concentration at the macula densa and possibly local angiotensin II production remains unclear. Another factor that could attenuate the TGF response in the models used is the natriuretic peptide ANP. It has been shown in micropuncture experiments that ANP reduces the responsiveness of TGF (Briggs et al., 1982; Huang and Cogan, 1987). Since all models used in this study involve high salt intake and/or hypervolemia, it is near at hands to speculate that ANP plasma levels are elevated. We did not determine plasma ANP concentrations in our mice, however cardiac ANP mRNA abundance was 4 to 12-fold elevated in the respective models compared to untreated wildtype mice (data not shown), indicating stimulation of ANP synthesis. Although high ANP levels might contribute to unresponsiveness of the TGF mechanism in some of our models, the fact that EMPA did not reduce hyperfiltration in global GC-A KO mice, which completely lack ANP signaling, argues against a general critical involvement of ANP in this process.

Undoubtedly, it is possible that EMPA exerts its protective potential via unknown mechanisms that are simply not triggered by the UNx/DOCA/salt model in mice. In this context, it should be mentioned that EMPA induced significant diuresis only in the models without DOCA treatment (Table 1; groups 4 and 5). In the DOCA groups (Table 1; groups 2 and 3), massive diuresis persisted even in H_2_0 mice without EMPA treatment. The interindividual variance of urine volumes in these groups corresponds approximately to the magnitude of the observed diuresis effect of EMPA in groups 4 and 5. It is therefore possible that diuretic effects of EMPA, corresponding trends are recognizable, in the DOCA groups do not reach the statistical significance level due to high data variability. Regardless of the effects in the DOCA groups, the data from groups 4 and 5, in which a clear diuretic effect of EMPA is evident, show that EMPA-induced diuresis is not the key renoprotective factor. Since SGLT2 inhibitors induce natriuresis it is near at hands to speculate that they should reduce extracellular volume. However, the pronounced diuresis in group 5 (GC-A KO without DOCA and salt) did not lead to a decrease in plasma volume. Because plasma volume was not determined in the other groups, the question of whether EMPA has an effect on plasma volume in any of the models cannot be answered, which is a limitation of our study. Also, for the interpretation of the results, knowledge of the effects of EMPA on blood pressure and ketone bodies formation in the individual groups would be very helpful. However, since these parameters were not measured in our study, these important points cannot be answered.

As seen in other rodent studies (Kapoor et al., 2015; Rajasekeran et al., 2018; Castoldi et al., 2020; Yamato et al., 2020; Castoldi et al., 2021), SGLT2 inhibition caused significant or at least a strong trend towards renal hypertrophy in our animal models under hyperfiltrating conditions, but also in healthy kidneys. Whether the weight gain of the kidney is due to expansion of cell volume or edema formation and which parts of the nephron experience cell growth cannot be answered at this point and requires further investigation.

Besides their nephroprotective effects, SGLT2 inhibitors reduce hospitalization rates and deaths form cardiovascular causes in diabetic and non-diabetic patients (McMurray et al., 2019; Packer et al., 2020). Similar to the nephroprotective effects the underlying mechanisms of cardioprotection are unclear and appear to be pleiotropic. Besides systemic effects such as blood pressure reduction, a positive influence of improved renal function on the cardiovascular system and direct cardiac effects of SGLT2 inhibitors have been suggested (Lopaschuk and Verma, 2020; Silva Dos Santos et al., 2020; Vallon and Verma, 2021). Since DOCA/salt treatment (Lother et al., 2016; Cao et al., 2019) as well as genetic deletion of the natriuretic peptide receptor GC-A (Kuhn et al., 2002; Kilic et al., 2005; Nakagawa et al., 2016) not only result in renal damage but also in cardiac hypertrophy and fibrosis, expression levels of marker genes for hypertrophy (BNP) and fibrosis (α-smooth muscle actin, TGF-β, collagen types 1 and 3) were determined in the respective models. As shown in Supplementary Figure S1 a non-significant trend towards reduced BNP and α-smooth muscle actin gene abundance was detected in EMPA treated animals, while TGF-β and collagen expression levels were completely unaffected by EMPA, indicating that EMPA does not affect cardiac fibrosis in these disease models.

Taken together, the data from several murine models of non-diabetic hyperfiltration, hypervolemia and hypertension suggest that nephroprotection by the SGLT2 inhibitor empagliflozin requires a functional TGF mechanism to reduce chronic hyperfiltration and protect animals from glomerular damage and albuminuria. As this is not the case in the murine models used in this study, other murine kidney disease models are needed for further investigations to definitely unravel the nephroprotective cellular mechanisms of SGLT2 inhibition in non-diabetic CKD.

## Data Availability

The raw data supporting the conclusions of this article will be made available by the authors, without undue reservation.

## Abbreviations

BSA, bovine serum albumin; CKD, chronic kidney disease; DOCA, deoxycorticosterone acetate; EMPA, empagliflozin; FITC, fluorescein-isothiocyanate; GFR, glomerular filtration rate; KO, knockout; TGF, tubuloglomerular feedback; UNx, unilateral nephrectomy; WT, wildtype.

## References

[B1] Abbas N. A. T. El Salem A. Awad M. M. (2018). Empagliflozin, SGLT2 Inhibitor, Attenuates Renal Fibrosis in Rats Exposed to Unilateral Ureteric Obstruction: Potential Role of Klotho Expression. Naunyn Schmiedebergs Arch. Pharmacol. 391 (12), 1347–1360. 10.1007/s00210-018-1544-y 30090949

[B2] Brenner B. M. Lawler E. V. Mackenzie H. S. (1996). The Hyperfiltration Theory: a Paradigm Shift in Nephrology. Kidney Int. 49 (6), 1774–1777. 10.1038/ki.1996.265 8743495

[B3] Brenner B. M. Troy J. L. Daugharty T. M. Deen W. M. Robertson C. R. (1972). Dynamics of Glomerular Ultrafiltration in the Rat. II. Plasma-Flow Dependence of GFR. Am. J. Physiol. 223 (5), 1184–1190. 10.1152/ajplegacy.1972.223.5.1184 4654351

[B4] Briggs J. P. Steipe B. Schubert G. Schnermann J. (1982). Micropuncture Studies of the Renal Effects of Atrial Natriuretic Substance. Pflugers Arch. 395 (4), 271–276. 10.1007/bf00580789 7155801

[B5] Cao H. J. Fang J. Zhang Y. L. Zou L. X. Han X. Yang J. (2019). Genetic Ablation and Pharmacological Inhibition of Immunosubunit β5i Attenuates Cardiac Remodeling in Deoxycorticosterone-Acetate (DOCA)-salt Hypertensive Mice. J. Mol. Cel Cardiol. 137, 34–45. 10.1016/j.yjmcc.2019.09.010 31629736

[B6] Cassis P. Locatelli M. Cerullo D. Corna D. Buelli S. Zanchi C. (2018). SGLT2 Inhibitor Dapagliflozin Limits Podocyte Damage in Proteinuric Nondiabetic Nephropathy. JCI Insight 3 (15), e98720. 10.1172/jci.insight.98720 PMC6129124 30089717

[B7] Castoldi G. Carletti R. Ippolito S. Colzani M. Barzaghi F. Stella A. (2020). Renal Anti-fibrotic Effect of Sodium Glucose Cotransporter 2 Inhibition in Angiotensin II-dependent Hypertension. Am. J. Nephrol. 51 (2), 119–129. 10.1159/000505144 31910407

[B8] Castoldi G. Carletti R. Ippolito S. Colzani M. Barzaghi F. Stella A. (2021). Sodium-glucose Cotransporter 2 Inhibition Prevents Renal Fibrosis in Cyclosporine Nephropathy. Acta Diabetol. 58 (8), 1059–1070. 10.1007/s00592-021-01681-2 33760995 PMC8272713

[B9] Chang Y. K. Choi H. Jeong J. Y. Na K. R. Lee K. W. Lim B. J. (2016). Dapagliflozin, SGLT2 Inhibitor, Attenuates Renal Ischemia-Reperfusion Injury. PLoS One 11 (7), e0158810. 10.1371/journal.pone.0158810 27391020 PMC4938401

[B10] Cherney D. Z. I. Dekkers C. C. J. Barbour S. J. Cattran D. Abdul Gafor A. H. Greasley P. J. (2020). Effects of the SGLT2 Inhibitor Dapagliflozin on Proteinuria in Non-diabetic Patients with Chronic Kidney Disease (DIAMOND): a Randomised, Double-Blind, Crossover Trial. Lancet Diabetes Endocrinol. 8 (7), 582–593. 10.1016/s2213-8587(20)30162-5 32559474

[B11] Cooper M. E. Inzucchi S. E. Zinman B. Hantel S. von Eynatten M. Wanner C. (2019). Glucose Control and the Effect of Empagliflozin on Kidney Outcomes in Type 2 Diabetes: An Analysis from the EMPA-REG OUTCOME Trial. Am. J. Kidney Dis. 74 (5), 713–715. 10.1053/j.ajkd.2019.03.432 31255334

[B12] Demerath T. Staffel J. Schreiber A. Valletta D. Schweda F. (2014). Natriuretic Peptides Buffer Renin-Dependent Hypertension. Am. J. Physiol. Ren. Physiol. 306 (12), F1489–F1498. 10.1152/ajprenal.00668.2013 24717731

[B13] Denic A. Mathew J. Lerman L. O. Lieske J. C. Larson J. J. Alexander M. P. (2017). Single-Nephron Glomerular Filtration Rate in Healthy Adults. N. Engl. J. Med. 376 (24), 2349–2357. 10.1056/NEJMoa1614329 28614683 PMC5664219

[B14] Fattah H. Layton A. Vallon V. (2019). How Do Kidneys Adapt to a Deficit or Loss in Nephron Number. Physiology (Bethesda) 34 (3), 189–197. 10.1152/physiol.00052.2018 30968755 PMC6734068

[B15] Grempler R. Thomas L. Eckhardt M. Himmelsbach F. Sauer A. Sharp D. E. (2012). Empagliflozin, a Novel Selective Sodium Glucose Cotransporter-2 (SGLT-2) Inhibitor: Characterisation and Comparison with Other SGLT-2 Inhibitors. Diabetes Obes. Metab. 14 (1), 83–90. 10.1111/j.1463-1326.2011.01517.x 21985634

[B16] Hayslett J. P. Kashgarian M. Epstein F. H. (1968). Functional Correlates of Compensatory Renal Hypertrophy. J. Clin. Invest. 47 (4), 774–799. 10.1172/jci105772 5641618 PMC297228

[B17] Heerspink H. J. Desai M. Jardine M. Balis D. Meininger G. Perkovic V. (2017). Canagliflozin Slows Progression of Renal Function Decline Independently of Glycemic Effects. J. Am. Soc. Nephrol. 28 (1), 368–375. 10.1681/asn.2016030278 27539604 PMC5198289

[B18] Heerspink H. J. L. Stefánsson B. V. Correa-Rotter R. Chertow G. M. Greene T. Hou F. F. (2020). Dapagliflozin in Patients with Chronic Kidney Disease. N. Engl. J. Med. 383 (15), 1436–1446. 10.1056/NEJMoa2024816 32970396

[B19] Huang C. L. Cogan M. G. (1987). Atrial Natriuretic Factor Inhibits Maximal Tubuloglomerular Feedback Response. Am. J. Physiol. 252 (5 Pt 2), F825–F828. 10.1152/ajprenal.1987.252.5.F825 2953251

[B20] Kapoor S. Rodriguez D. Riwanto M. Edenhofer I. Segerer S. Mitchell K. (2015). Effect of Sodium-Glucose Cotransport Inhibition on Polycystic Kidney Disease Progression in PCK Rats. PLoS One 10 (4), e0125603. 10.1371/journal.pone.0125603 25927597 PMC4416041

[B21] Kilic A. Velic A. De Windt L. J. Fabritz L. Voss M. Mitko D. (2005). Enhanced Activity of the Myocardial Na+/H+ Exchanger NHE-1 Contributes to Cardiac Remodeling in Atrial Natriuretic Peptide Receptor-Deficient Mice. Circulation 112 (15), 2307–2317. 10.1161/circulationaha.105.542209 16216978

[B22] Kim S. Jo C. H. Kim G. H. (2019). Effects of Empagliflozin on Nondiabetic Salt-Sensitive Hypertension in Uninephrectomized Rats. Hypertens. Res. 42 (12), 1905–1915. 10.1038/s41440-019-0326-3 31537914 PMC8075936

[B23] Kohan D. E. Fioretto P. Johnsson K. Parikh S. Ptaszynska A. Ying L. (2016). The Effect of Dapagliflozin on Renal Function in Patients with Type 2 Diabetes. J. Nephrol. 29 (3), 391–400. 10.1007/s40620-016-0261-1 26894924

[B24] Kuhn M. Holtwick R. Baba H. A. Perriard J. C. Schmitz W. Ehler E. (2002). Progressive Cardiac Hypertrophy and Dysfunction in Atrial Natriuretic Peptide Receptor (GC-A) Deficient Mice. Heart 87 (4), 368–374. 10.1136/heart.87.4.368 11907014 PMC1767056

[B25] Livak K. J. Schmittgen T. D. (2001). Analysis of Relative Gene Expression Data Using Real-Time Quantitative PCR and the 2(-Delta Delta C(T)) Method. Methods 25 (4), 402–408. 10.1006/meth.2001.1262 11846609

[B26] Lopaschuk G. D. Verma S. (2020). Mechanisms of Cardiovascular Benefits of Sodium Glucose Co-transporter 2 (SGLT2) Inhibitors: A State-Of-The-Art Review. JACC Basic Transl Sci. 5 (6), 632–644. 10.1016/j.jacbts.2020.02.004 32613148 PMC7315190

[B27] Lopez M. J. Wong S. K. Kishimoto I. Dubois S. Mach V. Friesen J. (1995). Salt-resistant Hypertension in Mice Lacking the Guanylyl Cyclase-A Receptor for Atrial Natriuretic Peptide. Nature 378 (6552), 65–68. 10.1038/378065a0 7477288

[B28] Lother A. Fürst D. Bergemann S. Gilsbach R. Grahammer F. Huber T. B. (2016). Deoxycorticosterone Acetate/Salt-Induced Cardiac but Not Renal Injury Is Mediated by Endothelial Mineralocorticoid Receptors Independently from Blood Pressure. Hypertension 67 (1), 130–138. 10.1161/hypertensionaha.115.06530 26553231

[B29] Ma Q. Steiger S. Anders H. J. (2017). Sodium Glucose Transporter-2 Inhibition Has No Renoprotective Effects on Non-diabetic Chronic Kidney Disease. Physiol. Rep. 5 (7), e13228. 10.14814/phy2.13228 28364032 PMC5392518

[B30] Madunić I. V. Breljak D. Karaica D. Koepsell H. Sabolić I. (2017). Expression Profiling and Immunolocalization of Na+-D-Glucose-Cotransporter 1 in Mice Employing Knockout Mice as Specificity Control Indicate Novel Locations and Differences between Mice and Rats. Pflugers Arch. 469 (12), 1545–1565. 10.1007/s00424-017-2056-1 28842746 PMC5691098

[B31] McMurray J. J. V. Solomon S. D. Inzucchi S. E. Køber L. Kosiborod M. N. Martinez F. A. (2019). Dapagliflozin in Patients with Heart Failure and Reduced Ejection Fraction. N. Engl. J. Med. 381 (21), 1995–2008. 10.1056/NEJMoa1911303 31535829

[B32] Mitchell K. D. Navar L. G. (1988). Enhanced Tubuloglomerular Feedback during Peritubular Infusions of Angiotensins I and II. Am. J. Physiol. 255 (3 Pt 2), F383–F390. 10.1152/ajprenal.1988.255.3.F383 3414799

[B33] Nakagawa H. Somekawa S. Onoue K. Kumazawa T. Ueda T. Seno A. (2016). Salt Accelerates Aldosterone-Induced Cardiac Remodeling in the Absence of Guanylyl Cyclase-A Signaling. Life Sci. 165, 9–15. 10.1016/j.lfs.2016.09.011 27647418

[B34] Neal B. Perkovic V. Mahaffey K. W. de Zeeuw D. Fulcher G. Erondu N. (2017). Canagliflozin and Cardiovascular and Renal Events in Type 2 Diabetes. N. Engl. J. Med. 377 (7), 644–657. 10.1056/NEJMoa1611925 28605608

[B35] Nespoux J. Patel R. Hudkins K. L. Huang W. Freeman B. Kim Y. C. (2019). Gene Deletion of the Na+-Glucose Cotransporter SGLT1 Ameliorates Kidney Recovery in a Murine Model of Acute Kidney Injury Induced by Ischemia-Reperfusion. Am. J. Physiol. Ren. Physiol 316 (6), F1201–f1210. 10.1152/ajprenal.00111.2019 PMC6620597 30995111

[B36] Packer M. Anker S. D. Butler J. Filippatos G. Pocock S. J. Carson P. (2020). Cardiovascular and Renal Outcomes with Empagliflozin in Heart Failure. N. Engl. J. Med. 383 (15), 1413–1424. 10.1056/NEJMoa2022190 32865377

[B37] Packer M. (2021). Mechanisms Leading to Differential Hypoxia-Inducible Factor Signaling in the Diabetic Kidney: Modulation by SGLT2 Inhibitors and Hypoxia Mimetics. Am. J. Kidney Dis. 77 (2), 280–286. 10.1053/j.ajkd.2020.04.016 32711072

[B38] Perkovic V. de Zeeuw D. Mahaffey K. W. Fulcher G. Erondu N. Shaw W. (2018). Canagliflozin and Renal Outcomes in Type 2 Diabetes: Results from the CANVAS Program Randomised Clinical Trials. Lancet Diabetes Endocrinol. 6 (9), 691–704. 10.1016/s2213-8587(18)30141-4 29937267

[B39] Perkovic V. Jardine M. J. Neal B. Bompoint S. Heerspink H. J. L. Charytan D. M. (2019). Canagliflozin and Renal Outcomes in Type 2 Diabetes and Nephropathy. N. Engl. J. Med. 380 (24), 2295–2306. 10.1056/NEJMoa1811744 30990260

[B40] Rajasekeran H. Reich H. N. Hladunewich M. A. Cattran D. Lovshin J. A. Lytvyn Y. (2018). Dapagliflozin in Focal Segmental Glomerulosclerosis: a Combined Human-Rodent Pilot Study. Am. J. Physiol. Ren. Physiol. 314 (3), F412–f422. 10.1152/ajprenal.00445.2017 PMC5899226 29141939

[B41] Sabrane K. Kruse M. N. Fabritz L. Zetsche B. Mitko D. Skryabin B. V. (2005). Vascular Endothelium Is Critically Involved in the Hypotensive and Hypovolemic Actions of Atrial Natriuretic Peptide. J. Clin. Invest. 115 (6), 1666–1674. 10.1172/jci23360 15931395 PMC1136988

[B42] Scheen A. J. (2014). Pharmacokinetic and Pharmacodynamic Profile of Empagliflozin, a Sodium Glucose Co-transporter 2 Inhibitor. Clin. Pharmacokinet. 53 (3), 213–225. 10.1007/s40262-013-0126-x 24430725 PMC3927118

[B43] Schnermann J. (2015). Concurrent Activation of Multiple Vasoactive Signaling Pathways in Vasoconstriction Caused by Tubuloglomerular Feedback: a Quantitative Assessment. Annu. Rev. Physiol. 77, 301–322. 10.1146/annurev-physiol-021014-071829 25668021

[B44] Schnermann J. B. Traynor T. Yang T. Huang Y. G. Oliverio M. I. Coffman T. (1997). Absence of Tubuloglomerular Feedback Responses in AT1A Receptor-Deficient Mice. Am. J. Physiol. 273 (2 Pt 2), F315–F320. 10.1152/ajprenal.1997.273.2.F315 9277593

[B45] Schreiber A. Shulhevich Y. Geraci S. Hesser J. Stsepankou D. Neudecker S. (2012). Transcutaneous Measurement of Renal Function in Conscious Mice. Am. J. Physiol. Ren. Physiol. 303 (5), F783–F788. 10.1152/ajprenal.00279.2012 22696603

[B46] Sharma M. Sharma R. McCarthy E. T. Savin V. J. Srivastava T. (2017). Hyperfiltration-associated Biomechanical Forces in Glomerular Injury and Response: Potential Role for Eicosanoids. Prostaglandins Other Lipid Mediat. 132, 59–68. 10.1016/j.prostaglandins.2017.01.003 28108282 PMC5513797

[B47] Silva Dos Santos D. Polidoro J. Z. Borges-Júnior F. A. Girardi A. C. C. (2020). Cardioprotection Conferred by Sodium-Glucose Cotransporter 2 Inhibitors: a Renal Proximal Tubule Perspective. Am. J. Physiol. Cel Physiol. 318 (2), C328–c336. 10.1152/ajpcell.00275.2019 31721613

[B48] Song P. Huang W. Onishi A. Patel R. Kim Y. C. van Ginkel C. (2019). Knockout of Na+-Glucose Cotransporter SGLT1 Mitigates Diabetes-Induced Upregulation of Nitric Oxide Synthase NOS1 in the Macula Densa and Glomerular Hyperfiltration. Am. J. Physiol. Ren. Physiol. 317 (1), F207–f217. 10.1152/ajprenal.00120.2019 PMC6692722 31091127

[B49] Staffel J. Valletta D. Federlein A. Ehm K. Volkmann R. Füchsl A. M. (2017). Natriuretic Peptide Receptor Guanylyl Cyclase-A in Podocytes Is Renoprotective but Dispensable for Physiologic Renal Function. J. Am. Soc. Nephrol. 28 (1), 260–277. 10.1681/asn.2015070731 27153922 PMC5198264

[B50] Szekeres Z. Toth K. Szabados E. (2021). The Effects of SGLT2 Inhibitors on Lipid Metabolism. Metabolites 11 (2), 87. 10.3390/metabo11020087 33535652 PMC7912792

[B51] Thomas M. C. Cherney D. Z. I. (2018). The Actions of SGLT2 Inhibitors on Metabolism, Renal Function and Blood Pressure. Diabetologia 61 (10), 2098–2107. 10.1007/s00125-018-4669-0 30132034

[B52] Thomson S. C. Vallon V. (2021). Effects of SGLT2 Inhibitor and Dietary NaCl on Glomerular Hemodynamics Assessed by Micropuncture in Diabetic Rats. Am. J. Physiol. Ren. Physiol. 320 (5), F761–f771. 10.1152/ajprenal.00552.2020 PMC8174804 33645318

[B53] Traynor T. Yang T. Huang Y. G. Krege J. H. Briggs J. P. Smithies O. (1999). Tubuloglomerular Feedback in ACE-Deficient Mice. Am. J. Physiol. 276 (5), F751–F757. 10.1152/ajprenal.1999.276.5.F751 10330057

[B54] Vallon V. Thomson S. C. (2020). The Tubular Hypothesis of Nephron Filtration and Diabetic Kidney Disease. Nat. Rev. Nephrol. 16 (6), 317–336. 10.1038/s41581-020-0256-y 32152499 PMC7242158

[B55] Vallon V. Verma S. (2021). Effects of SGLT2 Inhibitors on Kidney and Cardiovascular Function. Annu. Rev. Physiol. 83, 503–528. 10.1146/annurev-physiol-031620-095920 33197224 PMC8017904

[B56] van Bommel E. J. M. Muskiet M. H. A. van Baar M. J. B. Tonneijck L. Smits M. M. Emanuel A. L. (2020). The Renal Hemodynamic Effects of the SGLT2 Inhibitor Dapagliflozin Are Caused by post-glomerular Vasodilatation rather Than Pre-glomerular Vasoconstriction in Metformin-Treated Patients with Type 2 Diabetes in the Randomized, Double-Blind RED Trial. Kidney Int. 97 (1), 202–212. 10.1016/j.kint.2019.09.013 31791665

[B57] Wang H. Garvin J. L. Carretero O. A. (2001). Angiotensin II Enhances Tubuloglomerular Feedback via Luminal AT(1) Receptors on the Macula Densa. Kidney Int. 60 (5), 1851–1857. 10.1046/j.1523-1755.2001.00999.x 11703603

[B58] Wanner C. Inzucchi S. E. Lachin J. M. Fitchett D. von Eynatten M. Mattheus M. (2016). Empagliflozin and Progression of Kidney Disease in Type 2 Diabetes. N. Engl. J. Med. 375 (4), 323–334. 10.1056/NEJMoa1515920 27299675

[B59] Wiviott S. D. Raz I. Bonaca M. P. Mosenzon O. Kato E. T. Cahn A. (2019). Dapagliflozin and Cardiovascular Outcomes in Type 2 Diabetes. N. Engl. J. Med. 380 (4), 347–357. 10.1056/NEJMoa1812389 30415602

[B60] Yamato M. Kato N. Kakino A. Yamada K. I. Inoguchi T. (2020). Low Dose of Sodium-Glucose Transporter 2 Inhibitor Ipragliflozin Attenuated Renal Dysfunction and Interstitial Fibrosis in Adenine-Induced Chronic Kidney Disease in Mice without Diabetes. Metabol. Open. 7, 100049. 10.1016/j.metop.2020.100049 33015603 PMC7520892

[B61] Yamazaki D. Konishi Y. Morikawa T. Kobara H. Masaki T. Hitomi H. (2020). Failure to Confirm a Sodium-Glucose Cotransporter 2 Inhibitor-Induced Hematopoietic Effect in Non-diabetic Rats with Renal Anemia. J. Diabetes Investig. 11 (4), 834–843. 10.1111/jdi.13205 PMC7378420 31880858

[B62] Zhang J. Wei J. Jiang S. Xu L. Wang L. Cheng F. (2019). Macula Densa SGLT1-NOS1-Tubuloglomerular Feedback Pathway, a New Mechanism for Glomerular Hyperfiltration during Hyperglycemia. J. Am. Soc. Nephrol. 30 (4), 578–593. 10.1681/asn.2018080844 30867247 PMC6442354

[B63] Zhang Y. Thai K. Kepecs D. M. Gilbert R. E. (2016). Sodium-Glucose Linked Cotransporter-2 Inhibition Does Not Attenuate Disease Progression in the Rat Remnant Kidney Model of Chronic Kidney Disease. PLoS One 11 (1), e0144640. 10.1371/journal.pone.0144640 26741142 PMC4711803

[B64] Zinman B. Wanner C. Lachin J. M. Fitchett D. Bluhmki E. Hantel S. (2015). Empagliflozin, Cardiovascular Outcomes, and Mortality in Type 2 Diabetes. N. Engl. J. Med. 373 (22), 2117–2128. 10.1056/NEJMoa1504720 26378978

