# Supplementary material for: Empagliflozin Reduces Renal Hyperfiltration in Response to Uninephrectomy, but Is Not Nephroprotective in UNx/DOCA/Salt Mouse Models
Source: Front Pharmacol. 2021 Dec 21;12:761855. doi: 10.3389/fphar.2021.761855 (PMC8724563; doi:10.3389/fphar.2021.761855)

Supplementary Material

**Material and Methods**

**Plasma empagliflozin quantification by LC-MS/MS analysis:**For determination of total empagliflozin concentrations, a 30 µl aliquot of plasma was mixed first with 5 µl of stable isotope-labeled internal standard solution empagliflozin-d4 (10 µM in methanol; Cay22369; Biomol, Hamburg, Germany) and then 150 µl 100 % methanol were added for protein precipitation. The precipitated samples were stored at -80°C overnight. For complete extraction, samples were centrifuged at 10000 g for 5 min at 4°C and supernatants were collected. The protein precipitates were washed with 100 µl 80 % methanol and respective supernatants were combined in a glass vial. Samples were dried using an infrared vortex vacuum evaporator (CombiDancer, Hettich AG, Baech, Switzerland) and reconstituted in 30 µl H_2_O. For determination of free empagliflozin in plasma, a 100 µl aliquot was ultra-filtered employing Centrifree centrifugal filters (Merck Millipore Ltd., Tullagreen, Ireland) with a 30 kDa molecular weight cutoff (centrifugation at 2000 g, for 20 min, RT). 45 µl filtrate was mixed with 5 µl internal standard and directly used for analysis.

HPLC-MS/MS analysis was performed using an Agilent 1200 Series HPLC system (Boeblingen, Germany) coupled to an API 4000 QTRAP mass spectrometer (AB SCIEX, Darmstadt, Germany) operating in positive ionization mode. A Kinetex C18 HPLC column (10 cm × 2.1 mm, 2.6 μm; Phenomenex, Aschaffenburg, Germany) equipped with a Security Guard column (C18, Phenomenex) was used. Gradient elution was performed with phase A consisting of 0.1% formic acid (FA) in water (v/v) and 100% acetonitrile as mobile phase B. The gradient for chromatographic separation started with 25 % B, increased to 80 % B at 2.50 min and further to 100% B at 5.80 min, stayed at 100 % B for 2 min, followed by a reconditioning of the column at the starting conditions until 12.50 min. The flow rate was set to 400 µl/min. The column was kept at 35 °C and an injection volume of 5 μl was used. Turbo ion spray source was operated employing the following parameters: gas 1 and 2: 50 psi and curtain gas: 10 psi. The ion spray voltage was set to 4500 V, the declustering potential to 81 V, the entrance potential to 10 V, the collision exit potential to 12 V, and the collision energy to 47 V. Detection was performed in multiple reaction monitoring (MRM) mode using the following ion transitions: m/z 451.1 [M+H]^±^ to m/z 71.05 for empagliflozin and m/z 455.1 to m/z 71.05 for the deuterated internal standard. Quantification was achieved using a calibration curve constructed from the area ratio of the unlabeled compound to the stable isotope-labeled standard (in MultiQuant 3.0.2 Software, version 3.0.8664.0, AB Sciex).

**Supplemental tables**

**Supplemental table 1. Custom primers used with SYBR Green qPCR**

| **Gene** | **Tissue** | **Forward (5´-3´)** | **Reverse (5´-3´)** |
| --- | --- | --- | --- |
| Fibronectin | kidney | AGGTTCGGGAAGAGGTTGTG | GGCGTAATGGGAAACCGTGT |
| α-Sma | kidney/heart | GAAGAGCTACGAACTGCCTGA | TTTCGTGGATGCCCGCTG |
| Collagen 1a1 | kidney | CTGACGCATGGCCAAGAAGA | ATACCTCGGGTTTCCACGTC |
| Rpl-32 | kidney | TGGAGGTGCTGCTGATGTG | CGTTGGGATTGGTGACTCTGA |
| Bnp | heart | CTGAAGGTGCTGTCCCAGAT | GTTCTTTTGTGAGGCCTTGG |

**Supplemental table 2. Probes used for TaqMan qPCR**

| **Gene** | **Tissue** | **Assay ID** |
| --- | --- | --- |
| Gapdh | heart | Mm99999915_g1 |
| Tgf-β | heart | Mm01178820_m1 |
| Collagen 1a1 | heart | Mm00801666_g1 |
| Collagen 3a1 | heart | Mm00802331_m1 |

**Supplemental figures**

**Supplemental figure 1: EMPA does not affect cardiac hypertrophy and fibrosis in UNx (DOCA/salt) kidney disease models.** Left ventricular mRNA expression levels of BNP (**A**, hypertrophy marker), α-smooth muscle actin, TGF-β, collagen 1 and 3 (**B-E**, fibrosis marker) were measured in Podo GC-A KO mice with UNx/DOCA/salt (n=8-9 animals per group), WT mice with UNx/DOCA/salt (n=6-7 animals per group) and GC-A KO mice with UNx (n=6-7 animals per group). Target gene mRNA expression was normalized to GAPDH mRNA levels and EMPA-induced changes in mRNA abundance are shown as percentage of the expression level in H_2_O animals of the respective genotype (100%). Bar charts show mean values (± SEM). EMPA, empagliflozin; UNx (U), unilateral nephrectomy; D, DOCA; S, high salt.


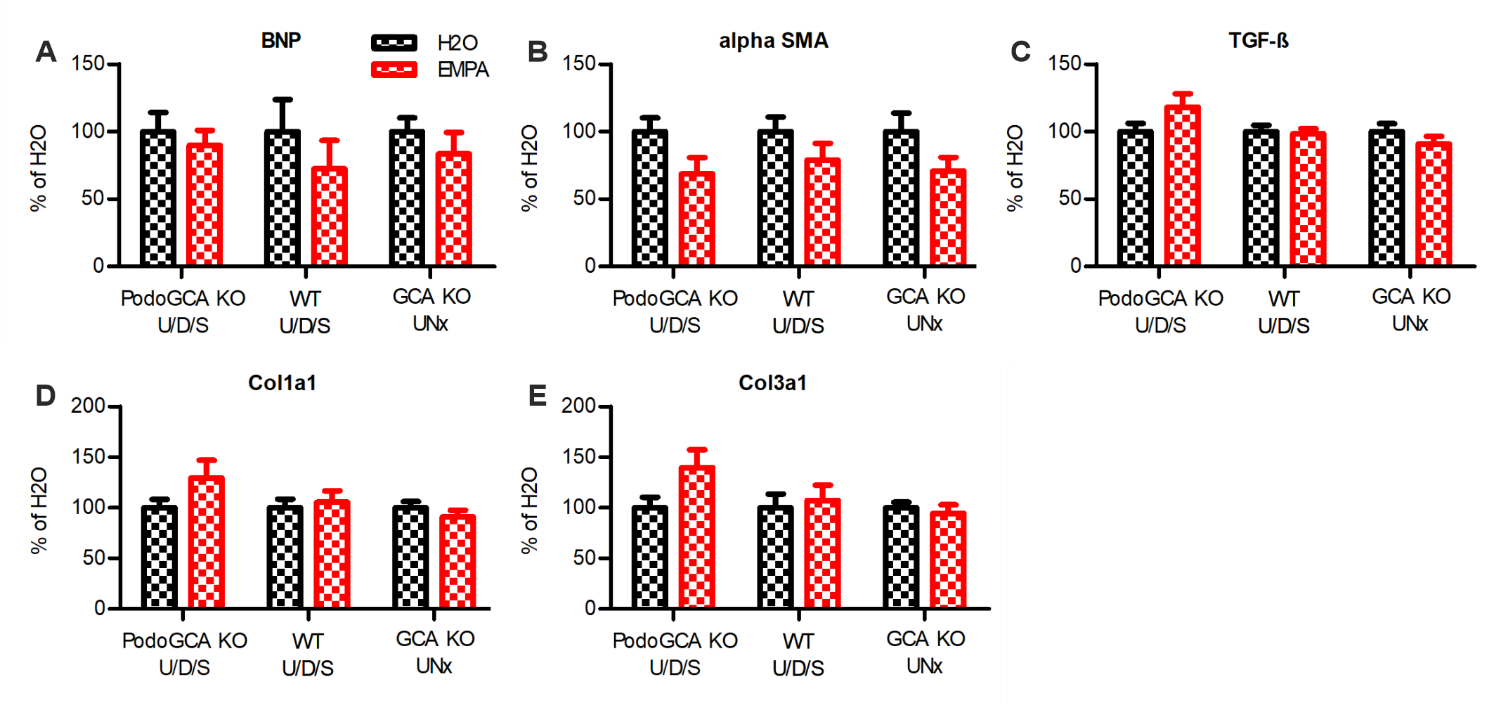

Supplement: Supplementary file 1 [file DataSheet1.docx]
